# Effectiveness of repeated photodynamic therapy in the elimination of intracanal *Enterococcus faecalis* biofilm: an in vitro study

**DOI:** 10.1007/s10103-017-2164-3

**Published:** 2017-02-10

**Authors:** Ewa Joanna Prażmo, Renata Alicja Godlewska, Agnieszka Beata Mielczarek

**Affiliations:** 1 Department of Conservative Dentistry, Medical University of Warsaw, Miodowa Street 18, 02-797 Warsaw, Poland; 2 Department of Bacterial Genetics, Faculty of Biology, University of Warsaw, Miecznikowa Street 1, 02-096 Warsaw, Poland

**Keywords:** Endodontic disinfection, *Enterococcus faecalis*, PDT, Photodynamic

## Abstract

The study aimed to investigate the effectiveness of photodynamic therapy in the elimination of intracanal *Enterococcus faecalis* biofilm and to analyse how a repeated light irradiation, replenishment of oxygen and photosensitiser affect the results of the photodynamic disinfecting protocol. After chemomechanical preparation, 46 single-rooted human teeth were infected with a clinical strain of *E. faecalis* and incubated for a week in microaerobic conditions. The experimental procedures included groups of single application of photodynamic therapy, two cycles of PDT, irrigation with 5.25% NaOCl solution and negative and positive control. The number of residing bacterial colonies in the root canals was determined based on the CFU/ml method. In the group of preparations irrigated with NaOCl, bacterial colonies were not observed. A single PDT eliminated 45% of the initial CFU/ml. Repeated PDT eradicated 95% of the intracanal bacterial biofilm. Photodynamic therapy has a high potential for the elimination of *E. faecalis* biofilm. There is a safe therapeutic window where photoinduced disinfection can be used as an adjuvant to conventional endodontic treatment, which remains the most effective.

## Introduction

Conventional endodontic therapy is performed to eliminate pathogenic microorganisms that are responsible for root canals’ infection. Because of the heterogeneity of the root canal system, complete disinfection and elimination of bacteria still remains a major challenge in endodontics. Mechanical preparation of root canal dentine, regardless of the tools applied, does not result in a uniform preparation and removal of the infected tissues [1]. The use of chemical agents during the treatment is obligatory to eliminate bacteria colonising dentinal tubules. However, common endodontic irrigants, like NaOCl, may be cytotoxic and neurotoxic when extruded in periapical tissue. Deep invasion of bacteria into dentinal tubules and the formation of biofilm are the main difficulties in the complete eradication of pathogenic microorganisms from the root canal system.

*Enterococcus faecalis* is commonly detected in persistent, asymptomatic endodontic infections with the formation of periapical lesions, when the revision of endodontic treatment is required [2]. Various virulence factors and survival mechanisms developed by these bacteria make it very difficult to eradicate by conventional methods of chemomechanical endodontic preparation [3]. The ability to compete with other microorganisms, the deep invasion of dentinal tubules, biofilm formation, resistance to nutritional depravation, and antibiotic resistance make this pathogen responsible for many endodontic failures [4]. Commonly used irrigant solutions are not able to completely eliminate infections caused by highly resistant *E. faecalis*. There is a need to develop new disinfection techniques to improve the results of conventional endodontic treatment.

Photodynamic therapy was created as an alternative cancer treatment protocol [5]. Its mechanism relies on correlations between three elements: photosensitive substance, light and oxygen [6]. Under aerobic conditions, a suitable wavelength of light activates the photosensitiser that results in the formation of a reactive oxygen species: superoxide, hydroxyl radicals and singlet oxygen. Activated photosensitiser and reactive oxygen species cause the disintegration of target tissues. Modern endodontics attempts to use these properties for alternative root canal disinfection [7]. Various types of photosensitisers have been subjected for observation. Toluidine blue, a basic thiazine metachromatic dye is one of the well-established photosensitisers used in PDT for targeting endodontic bacteria. Most of the photosensitising agents are activated by light between 630 and 700 nm. The aim of this study was to investigate the effectiveness of single and repeated photodynamic therapy in the elimination of *E. faecalis* biofilm and how the repetition of light irradiation, replenishment of oxygen and photosensitiser affects the results of photodynamic disinfection.

## Materials and methods

### *E. faecalis* strain isolation from the patient

*E. faecalis* is a Gram-positive coccus. It has developed complex defence mechanisms which make the eradication of this bacterium from the root canal system very difficult. There are also significant differences in virulence gene expression between the strains of *E. faecalis*. It was confirmed in relation to bacterial susceptibility to photodynamic therapy [8]. Silva et al. have observed that the efficacy of photodynamic antimicrobial chemotherapy on *E. faecalis* biofilms is strain dependent. In our study, *E. faecalis* was isolated form a root canal to strictly correlate the bacterial strain with a specific clinical situation and the therapeutic methods that might be applied in this specific condition.

A male, age 31, was admitted to the Department of Conservative Dentistry, Medical University of Warsaw for the purpose of dental treatment. A panoramic radiograph was performed for the assessment of the patient’s dentition and to establish a treatment plan. The lower left first molar required a revision of endodontic treatment. The tooth was asymptomatic; however, chronic periapical inflammation was diagnosed based on the presence of an osteolytic lesion on the radiograph. The patient was informed about the retreatment plan and accepted the proposal of microbiological analysis of intracanal material.

Tooth 36 was isolated with a rubber dam. All the roots were chemomechanically prepared to ISO #25 with the use of hand K-files and 0.9% NaCl as an irrigation solution. After the initial preparation, the root canal was filled with sterile saline solution and ISO #25 K file was used to perform scrubbing motions and to collect dentinal material for sampling. Sterile paper point ISO #25 was then introduced into the same space for 60 s to absorb the intracanal solution. Chemomechanical root canal preparation was continued with the use of standard irrigation protocol and was followed by warm gutta-percha condensation of the resulting space.

Microbiological material was transferred to tubes containing 3 ml of brain heart infusion broth (BHI) and was incubated for 24 h at 37 °C. After serial dilutions, aliquots of 100 μl were inoculated on plates with BHI (Oxoid) agar and on Slanetz and Bartley LAB-AGAR (Biocorp) plates (selective-differential medium for quantitative determination of enterococci, on which *E. faecalis* forms from dark pink to dark brown colour colonies).

### Strain identification

Chromosomal DNAs were isolated using a commercial kit and protocol (A&A Biotechnology, Poland). Polymerase chain reactions (PCRs) were performed with PrimeStar HS DNA Polymerase (TaKaRa) under standard conditions. The amplified 16S rRNA gene was obtained from isolate by PCR with the universal primers F27 (5′-AGAGTTTGATCMTGGCTCAG-3′) and R1492 (5′-TACGGYTACCTTGTTACGACTT-3′) [9] which are targeted to universally conserved regions and permit the amplification of an approximately 1500-bp fragment. Oligonucleotide synthesis and DNA sequencing were performed by Genomed S.A., Warsaw, Poland. The nucleotide sequences were analysed using BLAST against the nucleotide database on the NCBI website. The analysis revealed the highest identity, i.e., 99%, to the nucleotide sequence of the 16S rDNA gene of *E. faecalis* JF85 (GeneBank KT343158.1), thus identifying the taxonomic position of the strain as *E. faecalis*.

### Sample preparation

Forty-six extracted single-rooted, intact, mature, human teeth with a single canal were collected after the consent of the adult patients. Extractions were performed for periodontal or orthodontic reasons, and the obtained teeth were cleaned and stored in a 0.1% sodium azide solution. All samples were decoronated with diamond bur (Meisinger 859L.016) to acquire 15-mm-long roots. ISO #10 K-file was inserted into each of the canals until the instrument’s tip was visible at the apical foramen. The working length was established 1 mm short of the root length. All samples were instrumented with the use of hand K-files up to ISO #25. Further canal enlargement was performed to an apical size 40 (R40) using Reciproc rotary instruments (VDW, Munich, Germany). During chemomechanical preparation, a 2% NaOCl was used for irrigation. To remove the smear layer, each canal was finally rinsed with 17% EDTA. The root tips were sealed with glass ionomer cement, and the outer surface of the roots was covered with two layers of nail varnish to prevent reverse contamination of the dentinal tubules.

All samples were autoclaved at 121 °C for 15 min. Ten of them were randomly selected to assess the effectiveness of the sterilisation process as a negative control group and excluded from further experimental procedures. They were rinsed with sterile 0.9% NaCl, and a liquid from the canals was incubated on blood agar plates at 37 °C for 24 h. No bacterial growth was observed.

### Root canals’ contamination

A clinical strain of *E. faecalis* was cultured overnight in Tryptic Soy Broth at 37 °C, microaerobic conditions (5% O_2_, 10% CO_2_, 85% N_2_) without shaking and diluted to OD_600_ 0.7–0.8. By plating the suspension on BHI agar plates, the number of bacterial colonies was determined to be 1–3 × 10^8^ CFU/ml. Each sterile specimen was placed in a screw-cap plastic vial with 1 ml of TSB and 1 ml of bacterial suspension. The experimental units were incubated for 7 days to obtain biofilm formation (37 °C, microaerobic conditions). The TSB medium was replaced every 48 h with fresh TSB.

### Experimental protocol

All specimens were randomly divided into four equal groups *n* = 12. The positive control was used for the assessment of bacterial growth (Table 1). The roots were irrigated with 2 ml of 0.9% NaCl and subsequently the number of *E. faecalis* colonies was determined based on the CFU/ml method.

**Table 1:** Experimental groups evaluated in the study

Group	Number of specimens	Experimental procedure
0	10	Negative control group—evaluation of sterilisation process
Control	12	Positive control group—irrigation with 0.9% NaCl
PDT	12	Single PDT protocol
2PDT	12	Double PDT protocol
NaOCl	12	NaOCl irrigation—5 min

**Fig. 1:**
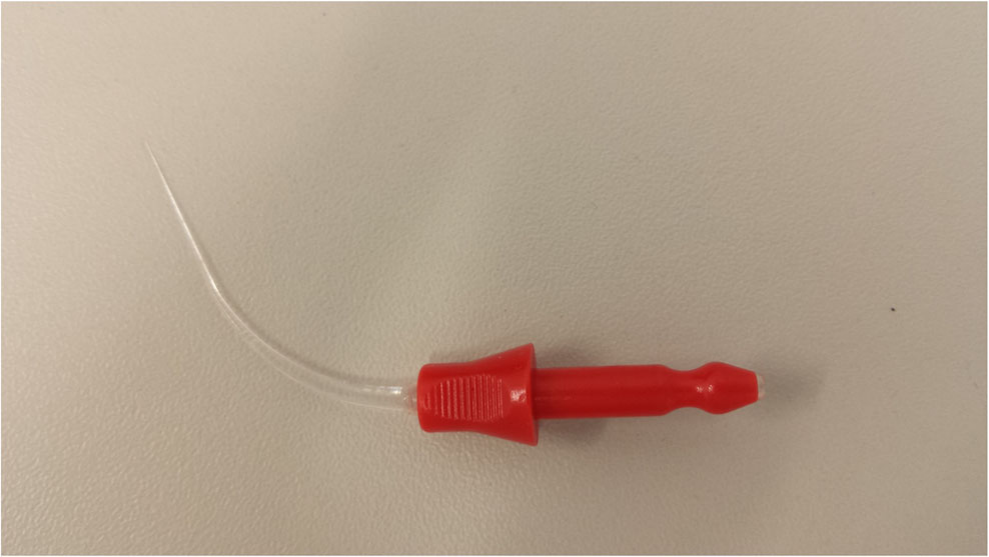
Laser light guide with rod-shaped irradiation for the root canal photodynamic therapy

Root canals from the PDT group were subjected to a single PDT protocol. Before the PDT procedure, each canal was irrigated with 2 ml of sterile 0.9% NaCl and dried with a paper point R40 (VDW, Munich, Germany). Toluidine blue solution (13–15 mg/ml) was used as a photosensitiser. Dye was placed into the canal for 2 min to allow accumulation in targeted tissues. Diode laser with a wavelength 635 nm was used as a light source for the PDT reaction with 120 mW, 12 J, 2-min parameters of irradiation. Laser radiation was delivered via special light guide (Fig. 1) that was introduced into the canal, 1 mm short of the apex in a pulse mode. To ensure equal irradiation of the infected dentine, the light guide was moved in a spiral fashion from the apex to the coronal part of the root and back to the apex. After each cycle of PDT, the application tip was wiped with a disinfecting solution, and the root canal was irrigated with 2 ml of 0.9% NaCl to evacuate the deactivated photosensitiser solution.

In the 2PDT group, photodynamic protocol, with the same parameters of laser irradiation, was repeated. There was a 2-min break between protocols and toluidine blue solution was renewed before the second laser application.

The last experimental NaOCl group underwent chemical disinfection with the use of 5.25% NaOCl. Each of the specimens was irrigated with 10 ml of this substance, which was subsequently left in the canals. The total time of contact with the dentine surface was 5 min. After chemical disinfection, the canals were irrigated with 2 ml of sterile 0.9% NaCl.

### Microbiological analysis

The *E. faecalis* present in the root canals’ contents were enumerated by plating. Following the experimental procedures, the canals were filled with sterile 0.9% NaCl. Hand K-files size 30 (VDW) were then introduced into canals to perform scrubbing movements for 15 s. Thereafter, sterile paper points R40 (VDW, Munich, Germany) were placed to collect microbiological material for analysis. After 60 s, they were transferred to Eppendorf-type probes with 1 ml of TSB. All probes were kept in a container filled with ice and, after 30 min, transported to the laboratory.

Tubes with paper points were agitated for 60 s. After tenfold serial dilutions, aliquots of 0.1 ml were plated on TSB agar medium and incubated at 37 °C for 72 h. Following the incubation period, bacterial colonies were counted. Based on the known dilutions, the actual number of *E. faecalis* colonies were calculated and given as *CFU*/*ml* (colony-forming units per millilitre).

### Statistical analysis

The resulting data were subjected to statistical analysis with the use of the Statistica 12 software. To verify the distribution of the parameters between research groups Shapiro–Wilk test was applied. In not related experimental groups without normal distribution, statistical calculations were based on the nonparametric Mann–Whitney *U* test (*p* = 0.05). The comparison of related groups (PDT and 2PDT) was performed with the use of Wilcoxon test (*p* = 0.05).

## Results

The results of the experiment and the number of *E. faecalis* colony-forming units after application of different disinfection methods are shown in Table 2. Based on the Mann–Whitney *U* test, a statistically significant reduction in the number of bacterial colonies in comparison with the control group was observed after second application of photodynamic therapy and irrigation with 5.25% NaOCl (*p* = 0.00). Single PDT protocol did not result in a significant change of intracanal *E. faecalis* inflammation (*p* = 0.052). Based on the Wilcoxon test used as a paired comparison test for the related experimental groups, statistically significant differences were observed between the PDT and the 2PDT group (*p* = 0,002).

**Table 2:** Count of *E. faecalis* CFU/ml after different disinfection protocols

Measurement conditions	Mean	SD	Median	Minimum	Maximum
Control	17.83 * 10^5^	24.12 * 10^5^	9.5 * 10^5^	3 * 10^5^	9 * 10^6^
PDT	9.83 * 10^5^	15.03 * 10^5^	2.5 * 10^5^	1 * 10^5^	5 * 10^6^
2PDT	9.67 * 10^4^	1.01 * 10^5^	4.5 * 10^4^	0.4 * 10^4^	3 * 10^5^
NaOCl	0	0		0	0

*SD* standard deviation

**Fig. 2:**
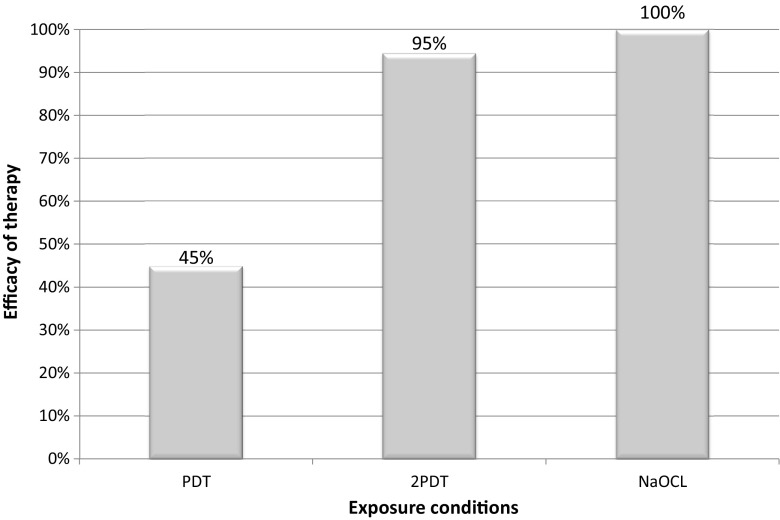
Effectiveness of different disinfection methods expressed as a percentage elimination of *Enterococcus faecalis* CFU

Under in vitro experimental conditions, the application of single photodynamic disinfection protocol allowed to reduce 45% of the initial *E. faecalis* colonies (Fig. 2). Two cycles of PDT were significantly more efficient and eradicated 95% of bacterial biofilm from infected root canals. In the group disinfected with NaOCl, no bacterial colonies were detected from the dentin surface, and the CFU value in the microbiological analysis of the obtained samples was 0

## Discussion

Complete endodontic therapy consists of mechanical preparation of the root canal and chemical disinfection of dentinal tubules. However, the conventional protocol of treatment is associated with the risk of leaving persistent bacteria within accessory canals, stenoses, and dentinal cracks. Final three-dimensional filling of the root canal may deprive bacteria an access to nutrients, but the risk of re-infection is always present. The aim of the described experiment was to investigate the efficacy of photodynamic therapy as a new method of root canal disinfection. *E. faecalis* was chosen as the test bacterial strain because it is one of the most resistant microorganisms, causing root canal infections. The study proved a high efficacy of repeated PDT protocol against bacterial biofilm with the use of toluidine blue as a photosensitiser and diode laser as a source of light.

The mechanism of photodynamic reaction is complicated. It is based on a mutual correlation of three factors: light, photosensitiser and oxygen. Each of the factors may be applied with various parameters, which makes it difficult to establish the most efficient therapeutic protocol for endodontic disinfection.

There is a difference in the susceptibility of microorganisms causing endodontic infection. *E. faecalis* as a Gram-positive coccus have a relatively porous membrane, built on a thick layer of peptidoglycan and lipoteichoic acid. This cellular morphology promotes a greater diffusion of photosensitiser into the bacterium. Gram-negative bacteria have an additional 10–15-nm-thick structural element, which is external to the peptidoglycan network and inhibits the penetration of many substances [10, 11]. It was confirmed that photosensitisers positively charged at physiological pH values, like toluidine blue, have a potential to photoinactivate both Gram-positive and Gram-negative bacterial cells [10]. TBO, a photosensitiser derived from phenothiazines, is compatible with an illumination wavelength of 620–650 nm. The maximum wavelength absorption occurs at 630 nm, which is essential for effective PDT protocol. The other important issue is that the effectiveness of photodisinfection may be dramatically reduced when TBO is washed out of the cell suspensions in the root canal before the application of light [12]. Demidowa et al. confirmed that the disinfecting results of PDT and the antimicrobial properties of different photosensitisers are correlated with the initial bacterial cell density in root canals. Higher pathogen cell density is associated with a significantly lower efficacy of PDT—the correlation is not linear. At the same time, different photosensitisers display different antibacterial potential against the same concentration of target cells. The variety of available photosensitisers and complexity of their application makes it difficult to compare the available results of performed experiments and to establish the most efficient therapeutic protocol.

The studies focused on photodynamic therapy as a method of endodontic disinfection agree that it cannot be applied without chemical irrigation. Each published research gives new observations and modifies photodynamic protocol to obtain better antibacterial properties. The light wavelength, applied in the course of photodynamic therapy, should be compatible with the photosensitiser used. The other light parameters, such as radiation density and power, also affect the mechanism of the photoinduced reaction. Studies focused on PDT application in tumour therapy confirmed that power exceeding 100 mW/cm^2^ reduces the potential to destruct pathologically changed cells [13]. High power leads to excessive consumption of oxygen in tissues and therefore to the depletion of the substrate for the production of reactive oxygen species. Inappropriate selection of light source parameters may additionally lead to gradual photobleaching of the photosensitiser [14].

The presented study was performed to investigate correlation between the repetition of photodynamic therapy and reduction of intracanal bacteria. The aim was to investigate how a repeated light irradiation after replenishment of oxygen and photosensitiser improves the results of the proposed photodynamic protocol. A single PDT cycle eliminated 45% of the bacterial colonies. After the second application of the photosensitiser and the subsequent irradiation cycle, the number of colonies was reduced to 5% of the initial number. The second dose of photosensitiser and light after replenishment of oxygen resulted in the statistically significant reduction of bacteria. Short irradiation cycle reduces the potential of photobleaching of the photosensitiser. The intermittent exposures to laser light allow for the replenishment of oxygen and application of the active photosensitiser. Our conclusions agree with the observations of Marinic et al. [15]. They have used eosin Y activated with the blue light at 450 mW/cm^2^ to eradicate *E. faecalis* biofilm. They have performed four PDT cycles of 4 min each after 10 min of photosensitiser accumulation in targeted tissues. The first cycle eliminated 42% of the bacterial colonies. The effectiveness of two PDT cycles was 88%, three cycles 93.5% and four cycles 96.2%. In our study, we have achieved similar disinfecting effects after 2 min incubation of photosensitiser and only two PDT cycles of 2 min each.

Our conclusions are consistent with the studies performed to compare the antibacterial effectiveness of PDT with different cycle durations. Xhevdet at al. [16] applied photodynamic therapy for 1, 3 and 5 min with the laser output power 100 mW and emission of 660 nm. The shortest cycle eliminated 54.35% of *E. faecalis* colonies from the extracted single-rooted teeth. Three minutes of irradiation reduced 69.45% of bacteria and the effectiveness of the longest PDT cycle was 71.59%. The elongation of laser irradiation improved the results of photodynamic therapy; however, the differences between the experimental groups have not been statistically significant. The 5-min cycle was much less effective than two cycles of total 4 min, applied in our experiment. Yildirim et al. [17] have also performed a study to compare the effect of different irradiation durations on the antimicrobial efficiency of PDT. The experimental groups were irradiated for 1, 2 and 4 min and there were no significant differences between them. The effectiveness of the applied photodynamic protocols was very high 99.8–99.9%; however, the authors did not reveal the power of laser irradiation and the concentration of photosensitiser, which makes it difficult to refer to the results.

The outcomes of the experiment confirm that photodynamic therapy is efficient in the reduction of *E. faecalis* bacterial biofilm. The in vitro experimental conditions did not allow for complete eradication of the infectious factor. However, the CFU/ml method, used in the study to determine the number of remaining bacterial colonies, has its restrictions. It does not measure *E. faecalis* colonies penetrating deep into dentinal tubules. It is impossible to determine quantitatively the number of bacteria remaining in deeper dentin layers after the application of different disinfection methods. In an aerobic environment rich in nutrients, *E. faecalis* penetrates the dentinal tubules to the mean depth of 1483.33 μm [18]. Under anaerobic conditions, the penetration depth decreases to 1166.66 μm, and the smallest measured depth of tubules’ colonisation is 620 μm in an anaerobic environment additionally deprived of nutrients [18]. NaOCl dentinal tubules’ penetration ability is significantly lower. In the study by Berutti et al., the depth was found to be 130 μm after the removal of the smear layer from the surface of the dentine [19]. Therefore, the absence of bacterial cells in the collected material after the experimental irrigation with NaOCl does not indicate a complete elimination of the infectious factor throughout dentinal tubules. Photodynamic therapy is a new treatment protocol in dentistry, and the number of corresponding studies is limited. Bumb et al. evaluated the depth of dentinal tubules at which PDT is effective in eliminating *E. faecalis* [20]. Scanning electron microscope images confirmed the absence of infection at the depth of 890–900 μm. These values indicate that photodynamic therapy shows significantly higher efficacy in eliminating *E. faecalis* from the depth of dentinal tubules than conventionally used NaOCl.

Irrigation with NaOCl presented the best antibacterial properties in the described study. During irrigation, the flow of liquid leads to the evacuation of damaged bacterial cells from root canals and increases penetration into dentinal tubules. Application of photosensitiser with additional ultrasonic vibration before the application of light might also be a good modification to improve results of photodynamic therapy. Ultrasonic waves have a potential to enhance the penetration of the photoactivated substance into the biofilm structure as it improves the efficacy of irrigation.

The safety of disinfection method is also an important issue. A high concentration of NaOCl is cytotoxic for the tissues surrounding the apical region of a tooth. George et al. [21] showed that the cytotoxicity of PTD is significantly lower than 5.25% NaOCl. Their study indicated that light-induced disinfection produces an insignificant effect on mammalian cells. Xu et al. [22] confirmed this thesis. According to them, photodynamic parameters, similar to those that may be applied in endodontic disinfection, have modest effects on osteoblasts and gingival fibroblasts, whereas sodium hypochlorite completely eliminates these cells.

## Conclusions

The results of the described in vitro experiment confirmed the high potential of photodynamic therapy in the elimination of *E. faecalis* biofilm. The cited studies explain mechanisms that affect the results of photoinduced disinfection in the endodontic treatment. These data indicate that there is a safe therapeutic window where PDT can be used against endodontic pathogens. It does not show toxicity against periapical tissues, effectively eliminates microorganisms organised in the biofilm structure and is not associated with the risk of resistance development, contrary to antibiotic therapy. Irrigation with NaOCl shows the best results in the reduction of dentine-colonising bacteria. Photodynamic therapy might be recommended as an adjuvant to conventional endodontic treatment, which remains the most effective antibacterial protocol.
